# Intra-arrest hypothermia during cardiac arrest: a systematic review

**DOI:** 10.1186/cc11235

**Published:** 2012-03-07

**Authors:** Sabino Scolletta, Fabio Silvio Taccone, Per Nordberg, Katia Donadello, Jean-Louis Vincent, Maaret Castren

**Affiliations:** 1 Department of Intensive Care, Erasme Hospital, Université libre de Bruxelles, Route de Lennik 808, 1070 Brussels, Belgium; 2 Department of Cardiology, Södersjukhuset, Karolinska Institute, Stockholm, Sweden; 3 Department of Clinical Science and Education, Section of Emergency Medicine, Södersjukhuset, Karolinska Institute, Stockholm, Sweden

## Abstract

**Introduction:**

Therapeutic hypothermia is largely used to protect the brain following return of spontaneous circulation (ROSC) after cardiac arrest (CA), but it is unclear whether we should start therapeutic hypothermia earlier, that is, before ROSC.

**Methods:**

We performed a systematic search of PubMed, EMBASE, CINAHL, the Cochrane Library and Ovid/Medline databases using "arrest" OR "cardiac arrest" OR "heart arrest" AND "hypothermia" OR "therapeutic hypothermia" OR "cooling" as keywords. Only studies using intra-arrest therapeutic hypothermia (IATH) were selected for this review. Three authors independently assessed the validity of included studies and extracted data regarding characteristics of the studied cohort (animal or human) and the main outcomes related to the use of IATH: Mortality, neurological status and cardiac function (particularly, rate of ROSC).

**Results:**

A total of 23 animal studies (level of evidence (LOE) 5) and five human studies, including one randomized controlled trial (LOE 1), one retrospective and one prospective controlled study (LOE 3), and two prospective studies without a control group (LOE 4), were identified. IATH improved survival and neurological outcomes when compared to normothermia and/or hypothermia after ROSC. IATH was also associated with improved ROSC rates and with improved cardiac function, including better left ventricular function, and reduced myocardial infarct size, when compared to normothermia.

**Conclusions:**

IATH improves survival and neurological outcome when compared to normothermia and/or conventional hypothermia in experimental models of CA. Clinical data on the efficacy of IATH remain limited.

## Introduction

Use of mild therapeutic hypothermia, or "targeted temperature management" as recently suggested [1], has been recommended in cardiac arrest (CA) patients since the publication of two randomized clinical trials in 2002, the results of which demonstrated a significant improvement in neurologically intact survival for comatose CA patients presenting with ventricular fibrillation (VF) or ventricular tachycardia (VT) [2,3]. Current guidelines suggest that mild therapeutic hypothermia should also be considered in patients presenting with other rhythms although this has been less well studied [4].

Although therapeutic hypothermia has been widely implemented [5], its benefits are still questioned and several issues remain unanswered, including the optimal time to initiate cooling. Animal data have indicated that early cooling after return of spontaneous circulation (ROSC) produces better brain function and neurological recovery than does normothermia, whereas delaying therapeutic hypothermia significantly limited these beneficial effects [6,7]. There are also experimental data suggesting that hypothermia initiated during cardiopulmonary resuscitation (CPR), that is, intra-arrest, is superior to cooling initiated after ROSC, both in terms of increased rates of successful CPR and improved survival [8-11]. Experimental investigations have also shown that intra-arrest therapeutic hypothermia (IATH) increases the success rate of defibrillation attempts in VF [12] and has beneficial effects on heart function, including improved left ventricular function and reduced myocardial infarct size [13]. Clinical investigations have shown that pre-hospital induction of therapeutic hypothermia is feasible [14], without major adverse events even when used intra-arrest [15], and may provide some additional benefits over delayed in-hospital cooling [16].

The objective of this systematic review of the literature was, therefore, to evaluate whether IATH affects survival and neurological and cardiac function in experimental and human CA.

## Materials and methods

### Search strategy

The following databases were searched up to 30 July 2011: PubMed (from 1966), EMBASE (from 1974), CINAHL (from 1982), the Cochrane Library (from 1974) and Ovid/Medline (from 1966). The search strategy used the following terms: "arrest" OR "cardiac arrest" OR "heart arrest" AND "hypothermia" OR "therapeutic hypothermia" OR "cooling". References from identified studies and relevant review articles were also searched for additional eligible citations. The search was limited to English publications and was conducted in accordance with the International Liaison Committee on Resuscitation (ILCOR) process of evidence evaluation [17].

### Study selection

Two authors (KD and FST) independently reviewed citations, abstracts and full-text articles to select eligible studies. We excluded: a) review articles; b) case reports; c) experimental studies other than animal studies (for example, cell culture, isolated organs); d) studies focusing on induced circulatory arrest (for example, for aortic arch surgery). During the selection process, we included all studies in which hypothermia was initiated before ROSC (that is, during CA); we then excluded studies in which IATH was not used alone (for example, together with cardiopulmonary bypass (CPB)). In case of disagreement, the eligibility of an article was decided by consensus among all authors (step 1).

For animal studies, we restricted studies to those that included a control group (either treated with normothermia or by conventional therapeutic hypothermia, that is, post-arrest therapeutic hypothermia (PATH), or another IATH group) and reported at least one of the following outcomes: a) overall mortality; b) brain function (neurological status, cerebral perfusion and/or metabolism); b) heart function (for example, rate of ROSC, characteristics of CPR, cardiac perfusion and performance) (step 2).

### Data abstraction and study quality

For each eligible study, two authors (SS and FST) independently abstracted data regarding: a) study design; b) characteristics of the study population; c) sample size; d) outcome measurements; and e) study quality. No attempt was made to re-analyze the data. Studies were classified by level of evidence (LOE) for studies with therapeutic interventions (Table 1) and quality (poor, fair or good) according to published definitions (step 3) [17]. Inter-observer agreement was calculated using percent agreement and the kappa statistic. The percent of agreement (kappa statistic) for each stage of selection was: step 1 = 89% (k, 0.87); step 2, 98% (k, 0.96); step 3 = 100% (k = 1).

**Table 1:** ILCOR levels of evidence for therapeutic interventions

**LOE 1**	Randomized controlled trials (RCTs) or meta-analyses of RCTs
**LOE 2**	Studies using concurrent controls without true randomization or meta-analyses of such studies
**LOE 3**	Studies using retrospective controls
**LOE 4**	Studies without a control group (for example, cases series)
**LOE 5**	Studies not directly related to the speciﬁc population (for example, different patients, animal models, mechanical models, and so on)

### Review end-points

The end-points of this study were to answer the following questions related to the use of IATH from the existing evidence:

1) What are the effects on mortality, brain and heart functions?

2) How should IATH be induced?

3) What are the potential adverse events?

## Results

### Search results

**Figure 1:**
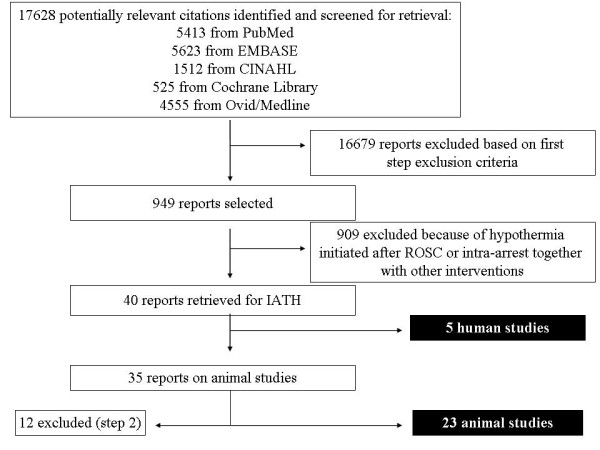
**Flow diagram of study selection process**.

The search retrieved a total of 17,628 citations (Figure 1). After application of the selection criteria, 28 articles were eligible for data abstraction: 23 were on experimental CA (LOE 5) and 5 on human CA (number of patients = 808). Among the clinical studies, one was a randomized controlled trial (RCT, LOE 1) [16], one a retrospective [18] and one a prospective [19] controlled study (LOE 3), and two were prospective studies without a control group (LOE 4) [15,20] (Table 2). Table 3 shows RCTs on IATH that are ongoing in the clinical setting. The characteristics of the included studies are shown in Tables 4, 5, 6.

**Table 2:** Evidence on intra-arrest hypothermia for human cardiac arrest

Quality of Evidence	Good	** Castrén, ABCD				
		* Castrén, E				
	Fair					
	Poor			* Garrett, A		
				** Garrett, BCD	* Bruel, E	
				** Callaway, E	* Kämärainen, E	
		**1**	**2**	**3**	**4**	**5**
		**Level of Evidence**				

Outcomes: A, return of spontaneous circulation; B, survival of event; C, survival to hospital discharge; D, intact neurological survival; E, reduction in temperature. Evidence, * supporting; ** neutral; *** opposing

**Table 3:** Ongoing clinical trials on intra-arrest therapeutic hypothermia in cardiac arrest

STATUS	NCT	STUDY
Active, recruiting	NCT01400373	PRINCESS: Prehospital Resuscitation Intra Nasal Cooling Effectiveness Survival Study
Active, recruiting	NCT00886184	HITUPPAC-BIO: Intra-arrest therapeutic hypothermia in pre-hospital cardiac arrest
Active, recruiting	NCT01413399	A pilot study of intra-arrest hypothermia in patients suffering non-traumatic out-of-hospital cardiac arrest
Active, recruiting	NCT01173393	Rapid Infusion of Cold Normal Saline During CPR for Patients With Non-VF Out-of-hospital Cardiac Arrest (RINSE)

**Table 4:** Summary of animal trials on intra-arrest hypothermia during cardiac arrest according to method of cooling

	Outcomes	IATH (%)	NT (%)	PATH (%)	Species	N	Arrest	Rhythm	No-flow (min)	HT method	IATH core temp (°C)	Time to ROSC (min)
**SYSTEMIC COOLING**												

**Abella **[**8**]	Survival (%, 3 days)	60	10*	10*	Mice	30	KCl	NA	8	Ice-water blanket	30	10 to 11
	GNO (%, 3 days)	60	10*	10*								
	ROSC (%)	100	100	100								

**Albaghdadi **[**35**]	ROSC (%)	78	45		Pigs	20	Asphyxia	NA	5	PFC-TLV	36	11 to 13
	CoPP (mmHg)	14	14									

**Boddicker **[**28**]	Survival (%, 24 hrs)	87	0*		Pigs	32	EC	VF	8	Surrounding the body with ice	33	12 to 18
	GNO (%, 24 hrs)	87	0*									
	ROSC (%)	87	0*									
	CoPP (mmHg)	15	10									

**Menegazzi **[**12**]	Survival (%, 20 min)	57	36		Pigs	28	EC	VF	8	ivfluid	34	13
	ROSC (%)	86	43*									
	CoPP (mmHg)	10	11									

**Nordmark **[**22**]	Survival (3-hrs)	80	100		Pigs	20	EC	VF	8	ivfluid	36	15 to 20
	ROSC (%)	90	100									

**Nordmark **[**25**]	Survival (%, 6 hrs)	100	100		Pigs	16	EC	VF	8	ivfluid	33	17
	LPR > 30 (n)	29	88*									
	SjO2 (%, 6 hr)	100	78*									
	ROSC (%)	100	100									

**Riter **[**10**]	Survival (%, 1 hr)	100	100		Pigs	24	EC	VF	11	PFC-TLV	35	22
	ROSC (%)	88	12*									
	CoPP (mmHg)	14	10									

**Shaffner **[**34**]	CBF (mL/100 g/min)	10	5*		Dogs	12	EC	VF	6	Ice	28	NA

**Staffey **[**15**]	ROSC (%)	82	27*		Pigs	33	EC	VF	11	PFC-TLV	33	22
	CoPP (mmHg)	10	10									

**Sterz **[**6**]	Survival (%, 3 days)	75	92	84	Dogs	36	EC	VF	10	Cold iv fluids	34	16 to 20
	GNO (%, 3 days)	50	8*	41						External cooling		
	NDS (3 days)	19	36*	22								
	CPP (mmHg)	55	50	51								
	ROSC (%)	75	92	84								
	CoPP (mmHg)	42	48	43								

**Xiao **[**24**]	Survival (%, 3 days)	100	100	100	Rats	30	Asphyxia	NA	5	Ice water	34	7
	GNO (%, 3 days)	100	40*	80								
	NDS (3 days)	1	20*	12*								
	ROSC (%)	100	100	100								

**Yannopoulos **[**13**]	ROSC (%)	100	66*	44	Pigs	45	CVO	VF	5	Intravascular catheter	33	20
	CoPP (mmHg)	21	21	20								
	LVEF (%)	32	23*	21*								

**Zhao **[**27**]	Survival (%, 7 days)	53	0*		Mice	45	KCl	NA	8	Blankets	30	10 to 11
	GNO (%, 3 days)	66	0*									
	ROSC (%)	93	80									
	dP/dt_max _(mmHg/s)	6000	3800*									

**SELECTIVE BRAIN COOLING**												

**Brader **[**26**]	Survival (24 hrs)	33	0		Dogs	12	EC	VF	4	Ice bags around the head	36	24
	NDS (24 hrs)	37	62*									
	ROSC (%)	82	82									

**Cho **[**32**]	Survival (hrs)	9.3	0.1*		Pigs	16	EC	VF/PEA	14	TNEC	36 to 37	19 to 29
	ROSC (%)	75	12*									
	CoPP (mmHg)	25	14*									

**Gelman **[**33**]	CPP (mmHg)	26	25		Pigs	14	EC	VF	6	Cooling cap placed around the head	38	NA
	CBF (mL/100 g/min)	16	18									
	CoPP (mmHg)	16	18									
	MBF (mL/100 g/min)	43	80*									

**Guan **[**29**]	Survival (%, 4-days)	100	29*	86	Pigs	24	EC	VF	10	TNEC	34-35	15 to 25
	NDS (4-days)	0	400*	0								

**Hagioka **[**23**]	Survival (%, 7-days)	100	100		Rats	14	CVO	VF	5	NFC	37	6
	GNO (%, 7-days)	100	100									
	CPP (mmHg)	40	34									
	HDS	33	71*									

**Jeung **[**21**]	Survival (%, 3 days)	72	72		Dogs	14	EC	VF	9	CF	35	12 to 15
	NDS (3 days)	18	42*									
	ROSC (%)	100	100									

**Tsai **[**30**]	Survival (%, 4 days)	100	25*	63	Pigs	24	EC	VF	10	TNEC	34	15 to 25
	ROSC (%)	100	88	88								
	CoPP (mmHg)	21	18	20								
	LVEF (%)	63	52*	51*								

**Tsai **[**31**]	Survival (%, 4 days)	100	29*		Pigs	16	EC	VF	10	TNEC	34	15 to 25
	NDS (%, 4 days)	0	400*									
	ROSC (%)	100	88									
	CoPP (mmHg)	21	18									
	LVEF (%)	63	53*									

**Wang **[**36**]	ROSC (%)	87	25*		Pigs	16	EC	VF	15	TNEC	34	20 to 30
	CoPP (mmHg)	25	16*									

CBF, cerebral blood flow; CF, carotid flush with cold saline fluids; CG, control group; CoPP, coronary perfusion pressure; CPP, cerebral perfusion pressure; CVO, coronary vascular occlusion; dP/dt, pressure/time ratio; EC, electrical current; GNO, good neurological outcome; HDS, histological deficit score; HT, hypothermia; IATH intra-arrest therapeutic hypothermia; KCl, potassium chloride; KT, catheter; LPR, lactate/pyruvate ratio; LVEF, left ventricular ejection fraction: MBF, myocardial blood flow; N, number; NA, not available; NDS, neurological deficit score; NFC, nasoparhyngeal cooling (saline); NT, normothermia; PATH, conventional (post-arrest) hypothermia; PFC-TLV, perfluorocarbon-based total liquid ventilation; ROSC, return of spontaneous circulation; SjO_2_, jugular bulb venous oxygen saturation; TNEC, trans-nasal evaporative cooling; VF, ventricular fibrillation; *, *P *< 0.05 vs. IATH

**Table 5:** Summary of animal trials that used different methods to induce intra-arrest hypothermia during cardiac arrest.

	Outcome	IATH (%)	IATH (%)
**Riter **[**10**]		PFC-TLV	iv fluids
		
	Survival (1-hr, %)	100	100
	ROSC (%)	88	25
	CoPP (mmHg)	14	8 *

**Yannopoulos **[**13**]		Intravascular catheter	iv fluids
		
	ROSC (%)	100	55 *
	CoPP (mmHg)	21	15 *
	LVEF (%)	32	17 *

**Yu **[**42**]		TNEC	iv fluids
		
	Survival (%, 4 days)	57	29
	ROSC (%)	100	29 *
	CoPP (mmHg)	23	12 *

CoPP, coronary perfusion pressure; LVEF, left ventricular ejection fraction; PFC-TLV, perfluorocarbon-based total liquid ventilation; ROSC, return of spontaneous circulation; TNEC, trans-nasal evaporative cooling; *, *P *< 0.05

**Table 6:** Summary of clinical trials on intra-arrest hypothermia (IATH) during cardiac arrest

	Setting	Method	Pts	CTRL	VF/VT (%)	Δt at admission	Δt vs CTRL	ROSC	Hospital admission	Survival all patients	Survival admitted pts
**Callaway **[**19**]	OHCA	Cranial cooling	22	Y (NT)	41	-0.02°C/min	NS	IATH 33% CTRL 0%	IATH 66% CTRL 15%	IATH 0% CTRL 0%	IATH 0% CTRL 0%
**Bruel **[**15**]	OHCA	Cold iv fluids	33	N	24	- 2.1°C	NA	61%	61%	12%	20%
**Kämärainen **[**20**]	OHCA	Cold iv fluids	17	N	59	-1.34°C	NA	71%	64%	6%	9%
**Castrén **[**16**]	OHCA	Trans-nasal	194	Y (PATH)	30	-1.3°C (tymp)	-0.7°C (core) - 1.3°C (tymp)	IATH 38% CTRL 43%	IATH 36% CTRL 42%	IATH 15% CTRL 13%	IATH 44% CTRL 31%
**Garrett **[**18**]	OHCA	Cold iv fluids	542	Y (NT)	22	NA	NA	IATH 37% CTRL 27%	IATH 28% CTRL 23%	IATH 13% CTRL 12%	IATH 44% CTRL 51%

Δt, difference in temperature; CTRL, control group; iv, intravenous; NA, not applicable/available; N, no; NS, not significant; NT, normothermia; OHCA, out-of-hospital cardiac arrest; PATH, conventional (post-arrest) hypothermia; Pts., patients; ROSC, return of spontaneous circulation; tymp, tympanic; VF, ventricular fibrillation; VT, ventricular tachycardia; Y, yes

### Effects on mortality

Sixteen animal and three human studies reported data on mortality.

#### a) IATH versus normothermia

Nine animal studies reported similar mortality rates in IATH and normothermia groups [6,10,12,21-26], and seven animal studies reported an improvement in survival rate in IATH-treated animals [8,27-32].

In an early human trial, external cranial IATH was investigated in 22 patients during out-of-hospital cardiac arrest (OHCA) [19], including 9 who received cranial cooling and 13 who did not; no patients survived to hospital discharge. A recent retrospective study compared the outcome of 208 OHCA patients treated with intra-arrest cold intravenous fluids to historical controls (*n *= 334) [18]. The use of IATH was not associated with increased overall survival to hospital admission (28% vs. 23%) or to discharge (13% vs. 12%).

#### b) IATH versus PATH

Two experimental studies reported improved survival with early IATH [8,13] and four [6,24,29,30] found no differences in mortality.

In the only RCT using IATH [16], OHCA patients were randomized, irrespective of their rhythm, to receive either trans-nasal evaporative cooling (TNEC, *n *= 96) or standard of care (*n *= 104, including PATH) during CPR. Overall survival rates were similar in the two groups (15% vs. 13%). Among patients admitted to the hospital, overall survival was increased, although not significantly, from 31 to 44% using IATH (*P *= 0.16). In the *post hoc *analysis, the sub-group of patients with time to CPR less than 10 minutes had an increased survival rate when treated by IATH (56% vs. 29%, *P *= 0.04). A non-significant 15% improvement in the survival rate was also observed in the small subgroup of VF patients.

### Effects on neurological outcome

Ten animal studies and one human study reported data on neurological outcomes.

#### a) IATH versus normothermia

In five studies, more animals in the intra-arrest cooling group had a good neurological outcome compared to normothermic animals [6,8,24,27,28]. In six studies, the neurological deficit score (NDS, where a score of 0 indicates no neurological deficit) was significantly lower in IATH than in normothermic animals [6,21,24,26,29,31]. Only two animal studies reported that IATH did not result in a better neurological outcome: In the first, all animals survived without neurological deficit at seen days after CA [23]; in the other, there were no differences in neurological outcome in terms of overall performance categories (OPC, 1 and 2 being good neurological outcome) or histological damage score (HDS) between IATH and normothermia groups [21]. Nevertheless, the percentage of injured neurons or the NDS was significantly lower in the IATH group compared with the normothermic group in these two studies [21,23].

#### b) IATH versus PATH

One experimental study [8] found a significant improvement in neurological status in animals treated with IATH compared to PATH, and another [24] showed a larger, although not statistically significant, number of animals with intact brain function in the IATH group.

In a human RCT, TNEC increased, although not significantly (*P *= 0.14), the intact neurological outcome rate from 21% to 34% when compared to a control group of patients admitted to hospital and cooled after CA [16]; these beneficial effects were more pronounced in patients with short time to CPR (43% vs. 17%, *P *= 0.03).

### Effects on brain perfusion and metabolism

Six animal studies reported data on brain perfusion and metabolism [6,22,23,25,33,34]. No human study has reported data on cerebral perfusion and/or metabolism during IATH.

#### a) IATH versus normothermia

Cerebral perfusion pressure (CPP) did not differ significantly between IATH and normothermia groups when selective brain cooling [23,33] or a combination of cold intravenous solutions and external cooling [6] were used. Cerebral blood flow (CBF) and cerebral oxygen extraction rate were similar during CPR and after ROSC in IATH and in normothermic animals [22,33]. Nevertheless, during CPR, hypothermic animals had higher blood flow in the caudate nucleus and the infra-tentorial regions than did normothermic animals [33], and cerebral oxygen uptake was significantly lower in hypothermic animals (44 vs. 69%, *P *= 0.02) at 45 minutes after reperfusion [33]. In a canine VF model of CA, IATH prevented the decrease in supratentorial blood flow and cerebral metabolic rate occurring during CPR at a CPP of 25 mmHg [34]. In another study, nasopharyngeal IATH resulted in a longer duration of high CBF at reperfusion when compared to normothermia [23]. Pigs treated with intra-arrest cooling had significantly higher values of venous jugular bulb saturation (SjO_2_), a surrogate for CBF, although CPP was similar during the study period [25]. Finally, cerebral metabolism was preserved during intra-arrest cooling as IATH prevented secondary increases in the lactate/pyruvate ratio (LPR) and glutamate levels when compared to normothermia [25].

#### b) IATH versus PATH

Only one animal study has reported data on brain perfusion and metabolism [6]: CPP was similar in IATH and PATH groups.

### Effects on cardiac function

Four animal studies have reported data on cardiac function [13,27,30,31]. No human study has specifically investigated heart function after CA when IATH therapy was used.

#### a) IATH versus normothermia

Left ventricular ejection fraction (LVEF) after CA was significantly increased by IATH compared to normothermia [13,30,31]. The ratio of infarction size to LV weight, a surrogate of degree of ischemic damage after coronary occlusion, was also noted to be significantly reduced by IATH when compared to normothermia [13]. Cardiac function evaluated by pressure-volume loops showed that early IATH resulted in improved cardiac output and contractility when compared to normothermia [27]. In another study, systolic and diastolic functions were both improved by cooling and the beneficial effects were more significant when hypothermia was initiated during CPR [31].

#### b) IATH versus PATH

In one animal study, cardiac function during IATH was compared to that during PATH [30]. Both IATH and PATH improved post-resuscitation myocardial dysfunction, but the beneficial effects were greatest with IATH [30]. In a second study, Yannopoulos *et al*. showed that intra-arrest cooling significantly improved LVEF and reduced the ischemic myocardial damage compared to delayed cooling [13].

### Effects on coronary perfusion pressure

Twelve animal studies reported data on coronary perfusion pressure (CoPP), defined as the difference between diastolic arterial pressure and central venous pressure, during CPR [6,10-13,28,30-33,35,36]. No human study has reported data on CoPP during IATH.

#### a) IATH versus normothermia

Ten studies showed that CoPP did not differ significantly between IATH and normothermia groups when different types of cooling were used [6,10-13,28,30,31,33,35]. One study reported a higher CoPP in the IATH group during the first three minutes of CPR, but not thereafter [10]. Two studies reported a higher CoPP before defibrillation when intra-arrest cooling was initiated using TNEC, compared to control normothermic animals [32,36].

#### b) IATH versus PATH

Three animal studies reported a similar CoPP in animals treated with IATH and those treated with PATH [6,13,30].

### Effects on return of spontaneous circulation (ROSC)

Eighteen animal studies [6,8,10-13,21,22,24-28,30-32,35,36] and three human studies [16,18,19] reported data on ROSC rate.

#### a) IATH versus normothermia

Ten experimental studies found no difference in ROSC rate between IATH and normothermia [6,8,21,22,24-27,30,31]. Albahgdadi *et al*. found a non-significant improvement in ROSC success with spontaneous circulation achieved in 78% of IATH and 45% of normothermic animals (*P *= 0.2) [35]. Seven animal studies reported an increase in ROSC rate with IATH [10-13,28,32,36].

In two clinical studies, IATH was associated with an increased ROSC rate compared to normothermia [18,19].

#### b) IATH versus PATH

Four experimental studies found no difference in ROSC rate between IATH and PATH [6,8,24,30]. One animal study noted that the ROSC rate was increased with IATH when compared to PATH [13]. In the human RCT using intra-arrest TNEC [16], patients receiving IATH had similar ROSC rates compared to the control group (38% vs. 43%).

### Effects on defibrillation attempts and epinephrine dose

Eleven animal studies reported data on the effects of IATH on duration and characteristics of CPR [6,10-13,21,28,30-32,36]. No human studies have specifically reported data on duration and characteristics of CPR during IATH.

#### a) IATH versus normothermia

In one study, there were no significant differences in the duration of CPR, the total dose of epinephrine administered during CPR or the number of counter-shocks given to reverse VF between animals that received IATH and those that remained normothermic [21]. Two other studies also found no difference in the number of defibrillation shocks required to achieve ROSC [10,32]. Sterz *et al*. [6] showed no differences in requirements of counter-shocks and the dose of epinephrine or in the duration of CPR between IATH- and normothermia-treated animals.

Seven animal studies reported variable effects of IATH on duration and characteristics of CPR [11-13,28,30,31,36]. In one, a somewhat smaller number of countershocks was required to achieve ROSC in IATH when compared to normothermia (9 vs. 17, *P *= 0.07) but the total dose of epinephrine required was lower as was the duration of CPR [31]. Tsai *et al*. reported that fewer defibrillation shocks were required to achieve ROSC in IATH compared to normothermia [30]; the total dose of epinephrine required to achieve ROSC was also lower in IATH as was the duration of CPR. In two studies, IATH achieved ROSC with a lower number of shocks but had a similar success rate as the normothermic group for initial shocks [11,12]. In a porcine model, the total amount of epinephrine used during the recovery period was three times less in the IATH than in the normothermia group [12]; however, the total number of defibrillation attempts did not differ between groups [11,12]. In another porcine model, there was no significant difference in the number of shocks needed (7 vs. 6.9) or in the success rate of initial shocks between IATH and normothermic animals (50 vs. 12%), but the duration of CPR was shorter in the IATH group [36]. Boddicker *et al*. reported that the first defibrillation was successful in 12% of normothermic compared to 75% of moderately cooled IATH animals (*P *= 0.04) [28]; the total number of delivered shocks was lower in IATH than in normothermic animals as was the number of re-fibrillations. Finally, Yannopoulos *et al*. reported that IATH was associated with fewer shocks to first ROSC, fewer total shocks and doses of epinephrine, and a shorter duration of CPR compared to normothermia [13]. No human studies have specifically reported data on duration and characteristics of CPR during IATH.

#### b) IATH versus PATH

Sterz *et al*. [10] showed no differences in requirements of counter-shocks and the dose of epinephrine or in the duration of CPR between IATH- and PATH-treated animals. In another study, IATH was associated with fewer shocks to first ROSC, fewer total shocks and doses of epinephrine, and a shorter duration of CPR compared to PATH [13].

### Effects of methods of inducing IATH

In animal studies, IATH has been induced by various techniques/devices, including ice packs, intravascular catheters, cold metal plates or total lung ventilation with perfluorocarbon (PFC). Some devices selectively cool the brain without affecting core body temperature. Few studies have compared the effects of these different devices on outcomes.

#### a) Effects of IATH method on outcomes

Three studies reported the effects of different types of IATH induction on mortality, brain or cardiac function after CA [10,13,37] (Table 5). LVEF was significantly increased by IATH induced by an intravascular system compared to intra-arrest cold fluids [13]; IATH using cold fluids resulted in a lower CoPP, a greater need for epinephrine and a longer duration of CPR. In a study by Riter *et al*. [10], cold fluids and PFC-TLV rapidly achieved target temperature, but ROSC was achieved more easily with PFC-TLV. In a model of prolonged CA, TNEC initiated during CPR improved the success of resuscitation compared with IATH induced by cold fluids; however, no difference in the survival rate was found [37]. The total number of countershocks and amount of epinephrine administered to achieve ROSC was significantly lower with IATH-TNEC than with cold fluids; the duration of CPR was also significantly reduced and CoPP higher when TNEC was used.

#### b) Effects of time of initiation of IATH

One experimental study investigated the role of early versus delayed induction of IATH [27]. Early IATH significantly improved survival and intact neurological outcome when compared to delayed IATH and was associated with improved cardiac output and contractility (evaluated by pressure-volume loops) [27].

#### c) Effects of target temperature during IATH

Only one animal study addressed the issue of the effects of different temperature levels during IATH on outcomes [28]. This study showed that animals suffering from prolonged VF-arrest had a better survival rate (87%) when treated with moderate (33°C) rather than mild (35°C, 37%, *P *< 0.001) or severe (30°C, 62%, *P *= 0.03) IATH, induced by external ice packs; ROSC rates were 87%, 37% and 62%, respectively. Moderate IATH had a higher successful VF termination rate (75%) with the first defibrillation attempt when compared to mild (50%) and severe (62%) IATH, although the number of refibrillation **{refibrillation} **episodes was similar among groups. Finally, the number of defibrillation attempts and the total energy delivered per animal were lower with deeper hypothermia.

### Adverse events

No adverse events associated with IATH were reported in the animal studies. In a human study of 16 patients who underwent IATH [15], rapid intravenous administration of cold fluids resulted in one case of pulmonary edema, which resolved after the prompt interruption of fluid infusion. In the Prince study, TNEC resulted in transient nasal whitening in 13 of 93 (14%) patients and epistaxis in three patients (serious in one patient with an underlying coagulopathy) [16]. Peri-orbital emphysema occurred in one patient and resolved spontaneously within 24 hours.

## Discussion

This systematic review revealed that: a) in experimental models of CA, IATH improves survival and neurological outcome when compared to normothermia and/or PATH; b) IATH improved ROSC rates and was associated with improved cardiac function, including better left ventricular function and reduced myocardial infarct size, when compared to normothermia; c) data on the efficacy of IATH in humans remain limited; d) IATH is feasible and is associated with few adverse events.

Therapeutic hypothermia has been shown to provide protective effects on brain and heart cells through different pathways. Hypothermic mechanisms providing myocardial protection have not been completely elucidated but include improved energy production during ischemia [38], inhibition of apoptosis [27], increased calcium sensitivity of myocytes [39], regulation of mitochondrial oxidative phosphorylation [38], attenuation of reactive oxygen species generation after ischemia-reperfusion [40], and preserved myocardial vascular autoregulation [41]. All these mechanisms translate into increased myocardial contractility [39,42], which should potentially be enhanced by IATH. Animal studies demonstrated a significant improvement in contractility when IATH was used in comparison with normothermia and PATH [13,27,30,31] and IATH also had the potential to reduce the infarction size after coronary vessel occlusion and CA [13]. Nevertheless, conflicting results have been reported between IATH and normothermia and/or PATH in terms of ROSC rate, duration of CPR, CoPP, total dose of epinephrine administered during CPR and defibrillation success rate [6,11,12,21,31]. Variations in the experimental models could explain, at least in part, these differences. A very short ischemic period is unlikely to produce sufficient cardiovascular alteration to make it possible to demonstrate any superiority of IATH over other therapies. In one study, time from CPR to ROSC was as short as two to three minutes [8] and in another, all the animals achieved ROSC within one minute after CPR was initiated [23]. The complexity and severity of the animal model, the different durations of VF, and the methods used to induce IATH (for example, systemic vs. selective brain) may also confound comparison of these studies. A further concern is related to the limited numbers of experimental animals used, as sometimes this restricted the power of statistical analyses [35]. Unfortunately, human studies have reported only data on the ROSC rate; in the RCT, TNEC resulted in similar ROSC rates to those seen in the control group [16], whereas in a retrospective study, IATH using cold fluids was associated with a significantly increased rate of ROSC compared to historical controls [18]. Further studies are needed to investigate myocardial function during IATH after human CA.

Therapeutic hypothermia may also preserve cerebral function after CA through inhibition of release of neurotransmitters, such as glutamate and dopamine, involved in brain damage [43]; preservation of the blood brain barrier [44]; protection of cerebral energy stores [45] and microcirculation [46]; and decrease in intracranial pressure and increase in CBF [47]. Importantly, cerebral reperfusion occurring after ROSC can also trigger the production of free radicals and other neurotoxic mediators, which may enhance anoxic damage to the brain [48]. Thus, the beneficial effects of therapeutic hypothermia after CA should be even more pronounced when cooling is initiated during the no-flow state and/or CPR before reperfusion. The positive effects of IATH on neurological outcome after experimental CA have been well-demonstrated. IATH affects brain perfusion and metabolism, as demonstrated by increased supra- and infra-tentorial CBF [23,33,34] and decreased cerebral oxygen uptake [25,33,34], thus preventing anaerobic metabolism and excito-toxicity [25]. All the experimental studies showed better neurological function in animals treated with IATH compared to normothermia or PATH, as well as a reduction in the number of injured neurons on histological brain examination. In the human RCT, IATH increased the intact neurological outcome rate, particularly in patients with a short no-flow time [16].

The combination of these beneficial effects of IATH on cardiac and cerebral functions should be expected to improve overall survival. However, IATH reduced mortality rates in only 7 of the 16 animal studies in which it was compared to normothermia [8,27-32] and 2 of the 6 studies in which it was compared to PATH [8,13]. Nevertheless, some of the negative studies had methodological limitations reducing the possibility of demonstrating any beneficial effects of IATH over standard treatment. In two of the studies, the observation period after CA was less than three hours [10,22], so that no effects on long-term outcome could be reported. In two studies, all animals survived after arrest because of a short no-flow and CPR period [23,24], this situation being far different from the clinical scenario of CA in humans. Two studies showed an improvement, although not statistically significant, in survival rate when IATH was used [26,35] and the authors acknowledged a limited sample size as the main reason for their findings.

Human data on the effects of IATH on mortality after CA are also scarce. External cranial cooling did not improve survival rates in OHCA compared to a control group without ice [19]; however, the reduction in tympanic temperature, a surrogate of brain temperature, was similar between groups. In a large retrospective database, intra-arrest cold intravenous fluids were not associated with increased overall survival; importantly, only 13% of the whole cohort received in-hospital therapeutic hypothermia. In the RCT using intra-arrest cooling [16], OHCA patients admitted to the hospital and receiving IATH had improved overall survival when compared to the control group although the difference was not statistically significant; these effects appeared to be even more beneficial in the sub-groups of patients with short no-flow time and VF.

If IATH is to be applied, it remains unclear which technique/device provides optimal cooling and protection. Speed of achieving target temperature in experimental studies was dependent on animal size and the duration of exposure to hypothermia during CA. These factors may limit the reproducibility of these data in the human setting. Moreover, although the neuroprotective effect of IATH may be related to how quickly the brain is cooled, systemic cooling is important for myocardial protection. Also, IATH using cold fluids resulted in a lower CoPP and decreased LVEF after CA [13]; the need for epinephrine and duration of CPR were also significantly longer than control animals, suggesting that post-ROSC myocardial dysfunction could be exacerbated by cold fluid loading. Cooling with an intravascular catheter, PFC-TLV or TNEC has been shown to improve ROSC rate, survival and intact neurological outcome when compared to cold fluid infusion [10,13,37]. Hence, although intravenous cold fluids have been shown to be effective and safe when applied after ROSC [2,14], the safety of large amounts of fluid during CPR may be questioned.

Other potential methods for IATH induction also have limitations when applied to the human setting. Internal cooling with catheters is invasive and not applicable in the field. Intrapulmonary PFC with TLV would not be easy to apply and may potentially exacerbate lung injury because of increased intrapulmonary pressure during ventilation [10,49]. Trans-nasal cooling devices spray a PFC fluid through a nasal catheter system into the nasal cavity and induce brain hypothermia via the cooled blood and by direct convection; body cooling occurs later. Further studies are needed to investigate the beneficial effects of this technique in the human setting as well as the occurrence of adverse events.

## Conclusion

The use of hypothermia has been associated with improved outcomes for survivors from CA; however, it has been suggested that the timing of the induced cooling may influence its beneficial effects. Experimental studies have shown that IATH can protect the heart against the ischemic processes occurring after CA and reduce the neuronal injury secondary to global ischemia. These beneficial effects seem to be significant when IATH is compared not only to normothermia, but also to PATH. Nevertheless, not all of these studies have reported similar conclusions, probably because of the different experimental conditions that have been used. Also, the evidence that IATH is superior to PATH in animal studies is more limited than the evidence comparing IATH to normothermia. Human data on IATH remain limited; however, several large cohort studies have suggested some beneficial effects of IATH on ROSC rates and neurological outcomes, especially if initiated within a short no-flow time. Selective brain cooling may have potential advantages in protecting the brain before reperfusion and has shown promising results in experimental and clinical studies; however, this technique may limit potentially beneficial effects of hypothermia on cardiac function and needs to be further evaluated in the human setting.

## Key messages

- IATH improves survival and neurological outcome when compared to normothermia and/or PATH in experimental models of CA.

- IATH prevents the decrease in cardiac function occurring after CA and maintains better cerebral perfusion and metabolism during CPR when compared to normothermia.

- Data on the efficacy of IATH in humans are still limited. One RCT showed better survival and neurological outcome in those patients receiving IATH with a no-flow time less than 10 minutes. Ongoing clinical trials will provide further data on the use of IATH in human CA.

- In the clinical setting, external cranial cooling, cold intravenous fluids and TNEC have been used to induce IATH. Few adverse events have been reported with these techniques.

## Abbreviations

CA: cardiac arrest; CBF: cerebral blood flow; CoPP: coronary perfusion pressure; CPB: cardiopulmonary bypass; CPP: cerebral perfusion pressure; CPR: cardiopulmonary resuscitation; HDS: histological damage score; IATH: intra-arrest therapeutic hypothermia; LOE: level of evidence; LVEF: left ventricular ejection fraction; NDS: neurological deficit score; OHCA: out-of-hospital cardiac arrest; OPC: overall performance categories; PATH: conventional, post-arrest therapeutic hypothermia; PFC: perfluorocarbon; RCT: randomized controlled trial; ROSC: return of spontaneous circulation; TNEC: trans-nasal evaporative cooling; VF: ventricular fibrillation; VT: ventricular tachycardia.

## Conflict of interests

FST received honoraria for lectures from Benechill, Inc. FST, MC, JLV and PN have conducted a study supported by Benechill, Inc. SC and KD have no conflicts of interest to declare.

## Authors' contributions

FST, MC and JLV conceived the study protocol and design. FST, SS and KD conducted the literature search, collected the data and performed the statistical analysis. FST, PN, JLV and MC participated in data interpretation. SS, FST and PN drafted the present manuscript, and JLV and MC revised the manuscript. All authors read and approved the final version of the manuscript.
